# Imaging timing after surgery for glioblastoma: an evaluation of practice in Great Britain and Ireland (INTERVAL-GB)- a multi-centre, cohort study

**DOI:** 10.1007/s11060-024-04705-3

**Published:** 2024-08-06

**Authors:** INTERVAL-GB Collaborative, Conor S Gillespie, Emily R Bligh, Michael TC Poon, Abdurrahman I Islim, Georgios Solomou, Melissa Gough, Christopher P Millward, Ola Rominiyi, Rasheed Zakaria, Stephen J. Price, Colin Watts, Sophie Camp, Thomas C Booth, Gerard Thompson, Samantha J Mills, Adam Waldman, Paul M. Brennan, Michael D Jenkinson, Hidayatul Abdullmalek, Suhaib Abualsaud, Gideon Adegboyega, Chinelo Afulukwe, Najma Ahmed, Michael Amoo, Abdelsalam Nedal Al-Sousi, Yahia Al-Tamimi, Ajitesh Anand, Neil Barua, Harsh Bhatt, Ion Boiangiu, Abbey Boyle, Christiaan Bredell, Talhah Chaudri, Jeremy Cheong, Ana Cios, David Coope, Ian Coulter, Giles Critchley, Harriet Davis, Paolo Jose De Luna, Nayan Dey, Bea Duric, Abdullah Egiz, Justyna O. Ekert, Chinedu Brian Egu, Jinendra Ekanayake, Anna Elso, Tomas Ferreira, Tom Flannery, Kwan Wai Fung, Rahul Ganguly, Sanay Goyal, Emily Hardman, Lauren Harris, Theodore Hirst, Kelvin Sunn Hoah, Sam Hodgson, Kismet Hossain-Ibrahim, Lena Mary Houlihan, Sami Squali Houssaini, Sadid Hoque, Dana Hutton, Mahnoor Javed, Neeraj Kalra, Siddarth Kannan, Efthymia Maria Kapasouri, Andrew Keenlyside, Kristy Kehoe, Bharti Kewlani, Prerna Khanna, Rosaline de Koning, Kunalika Sathish Kumar, Ashvin Kuri, Simon Lammy, Eunkyung Lee, Robert Magouirk, Andrew J Martin, Riccardo Masina, Ryan Mathew, Adele Mazzoleni, Patrick McAleavey, Gráinne McKenna, Daniel McSweeney, Saad Moughal, Mohammad Arish Mustafa, Engelbert Mthunzi, Armin Nazari, Trinh Ton Nu Ngoc, Shiva Nischal, Michael O’Sullivan, Jay J. Park, Anand S. Pandit, Jonathan Pesic Smith, Peter Peterson, Isaac Phang, Puneet Plaha, Shyam Pujara, George E. Richardson, Marwa Saad, Shinjan Sangal, Avani Shanbhag, Veekshith Shetty, Natalie Simon, Robert Spencer, Rosa Sun, Irtiza Syed, Jesvin Tom Sunny, Anca-Mihaela Vasilica, Daniel O’Flaherty, Arslan Raja, Daniele Ramsay, Renitha Reddi, Elena Roman, Ola Rominiyi, Dorina Roy, Omar Salim, Jeremiah Samkutty, Jashan Selvakumar, Thomas Santarius, Stuart Smith, Agbolahan Sofela, Edward Jerome St. George, Preethi Subramanian, Vaibhav Sundaresan, Kieron Sweeney, Boon Hoe Tan, Nicole Turnbull, Yuewei Tao, Lewis Thorne, Rebecca Tweedie, Anastasia Tzatzidou, Babar Vaqas, Sara Venturini, Kathrin Whitehouse, Peter Whitfield, Jack Wildman, Isabelle Williams, Karl Williams, Victoria Wykes, Tiffany Tze Shan Ye, Kelvin Sunn Yap, Mahir Yousuff, Asaad Zulfiqar, Neurology and Neurosurgery Interest Group (NANSIG), Soham Bandyopadhyay, Setthasorn Z. Y. Ooi, Abigail Clynch, Oliver Burton, Moritz Steinruecke, William Bolton, Alvaro Yanez Touzet, Hannah Redpath, Seong Hoon Lee, Joshua Erhabor, Orla Mantle, Conor S Gillespie, Emily S Bligh, British Neurosurgical Trainee Research Collaborative (BNTRC), Angelos Kolias, Julie Woodfield, Aswin Chari, Robin Borchert, Rory Piper, Daniel M. Fountain, Michael TC Poon, Abdurrahman I Islim

**Affiliations:** Cambridge, UK

**Keywords:** Glioblastoma, Neuroimaging, MRI, Neuro-oncology

## Abstract

**Purpose:**

Post-operative MRI is used to assess extent of resection, monitor treatment response and detect progression in high-grade glioma. However, compliance with accepted guidelines for follow-up MRI, and impact on management/outcomes is unclear.

**Methods:**

Multi-center, retrospective observational cohort study of patients with confirmed WHO grade 4 glioma (August 2018-February 2019) receiving oncological treatment. Primary objective: investigate follow-up MRI surveillance practice and compliance with recommendations from NICE (Post-operative scan < 72h, MRI every 3–6 months) and EANO (Post-operative scan < 48h, MRI every 3 months).

**Results:**

There were 754 patients from 26 neuro-oncology centers with a median age of 63 years (IQR 54–70), yielding 10,100 (median, 12.5/person, IQR 5.2–19.4) person-months of follow-up. Of patients receiving debulking surgery, most patients had post-operative MRI within 72 h of surgery (78.0%, *N* = 407/522), and within 48 h of surgery (64.2%, *N* = 335/522). The median number of subsequent follow-up MRI scans was 1 (IQR 0–4). Compliance with NICE and EANO recommendations for follow-up MRI was 52.8% (*N* = 398/754) and 24.9% (*N* = 188/754), respectively. On multivariable Cox regression analysis, increased time spent in recommended follow-up according to NICE guidelines was associated with longer OS (HR 0.56, 95% CI 0.46–0.66, *P* < 0.001), but not PFS (HR 0.93, 95% CI 0.79–1.10, *P* = 0.349). Increased time spent in recommended follow-up according to EANO guidelines was associated with longer OS (HR 0.54, 95% CI 0.45–0.63, *P* < 0.001) but not PFS (HR 0.99, 95% CI 0.84–1.16, *P* = 0.874).

**Conclusion:**

Regular surveillance follow-up for glioblastoma is associated with longer OS. Prospective trials are needed to determine whether regular or symptom-directed MRI influences outcomes.

**Graphical Abstract:**

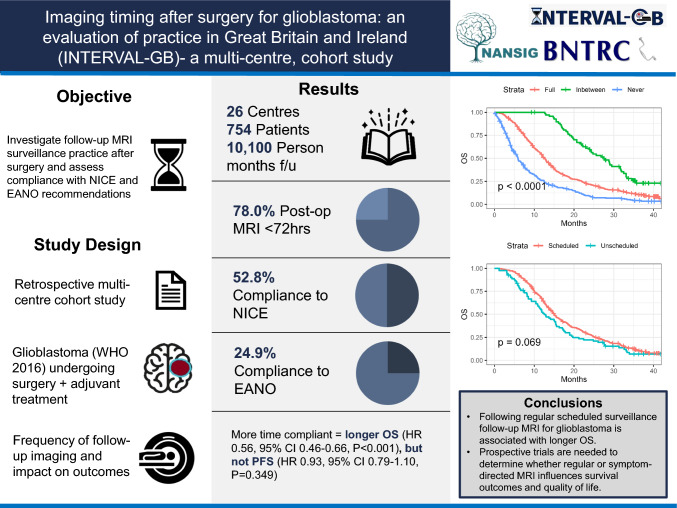

**Supplementary Information:**

The online version contains supplementary material available at 10.1007/s11060-024-04705-3.

## Introduction

Glioblastoma is the most common malignant primary brain tumor [1], with a median overall survival (OS) of 12–15 months (range 6–17 months), even with maximal treatment involving surgical resection and chemoradiotherapy [2–4]. Magnetic Resonance Imaging (MRI) is used to assess extent of tumour resection, monitor treatment response and detect disease progression [5]. Expert bodies such as the National Institute of Health and Care Excellence (NICE) and European Association of Neuro-Oncology (EANO) offer consensus-based guidelines, for post-operative scheduled surveillance MRI [5, 6]. Clinical deterioration may occur between surveillance intervals and will often prompt an unscheduled MRI scan.

There is a lack of evidence supporting an optimal imaging strategy, which has been highlighted in a recent Cochrane review [7]. The James Lind Alliance (JLA) Neuro-Oncology Priority Setting Partnership (PSP) identified “what is the effect on prognosis of interval scanning to detect tumor recurrence, compared to scanning on symptomatic recurrence” as a research priority [8]. The National Cancer Research Institute (NCRI) Brain Tumor group emphasised the need for appropriately powered studies on imaging timing, to address this evidence gap [9]. One single-center study proposed an optimal MRI schedule for molecular subtypes of glioblastoma using a parametric model of progression free survival (PFS), for (IDH) wild-type GBM. They suggested scanning every 7.4 weeks until 120 weeks post standard treatment, followed by scans every 27.6 weeks following a 22-week inflection period [10]. Multiple international surveys have demonstrated wide variation in clinical practice [11, 12], and the impact of post-operative imaging schedules on management and outcomes is unclear [5, 13–15]. We conducted the ImagiNg Timing aftER glioblastoma surgery: an eVALuation of practice in Great Britain and Ireland (INTERVAL-GB) study to determine the MRI surveillance practice in the UK, and if adherence to NICE/EANO guidelines is associated with OS and PFS.

The primary objective was to assess nationwide follow-up imaging schedules and indications according to NICE and EANO guidelines, with secondary objectives assessing the association of follow-up MRI schedules on OS and PFS.

## Methods

### Study design, setting and participants

This was a multicenter, retrospective cohort study of patients with newly-diagnosed glioblastoma undergoing surgery and oncological therapy. The study was conducted in 26 neuro-oncology centers in the UK and Ireland. The study protocol has previously been published and provides a detailed overview of the study design [16–18]. The analysis plan was changed once after protocol publication; changing the definition of ‘compliance’ to imaging guidelines from a binary variable (Compliant and noncompliant) to a continuous variable (time spent compliant), to acknowledge that many patients spent periods of time as compliant, and noncompliant, during the follow-up period. Local audit and Caldicott guardian approval was obtained at each unit before data collection could commence.

Data was captured consecutively on adult patients (aged ≥ 18 years) with a new histopathological diagnosis of glioblastoma (according to the diagnostic criteria at the time of diagnosis – the 2016 World Health Organisation [WHO] Classification) [19], who underwent surgery and any active oncological treatment between August 31, 2018, and February 1, 2019. Data collection took place between November 11, 2021, and May 22, 2022. Collaborators identified eligible patients by searching historic Multidisciplinary Team Meetings (MDTMs), histopathological and/or surgical records locally against the inclusion and exclusion criteria (Supplementary Table 1).

### Procedures

We used the Castor (Castor, NY, USA) online database to securely collect and store data. Data was collected by local investigators from a combination of the patient’s clinical, radiological and histopathological records. The variable domains consisted of baseline clinical and radiological variables, surgical and histopathological variables, adjuvant and supportive treatment details, follow-up MRI details and outcome measures. Follow-up MRI outcomes were determined by the overall scan report reported by a neuroradiologist at each participating center- and classified into three groups: stable, recurrence/further growth, and pseudoprogression. To be entered, reports had to be confirmed by MDTM agreement. The minimum data requirement for a case to be included was the baseline characteristics, surgery date, and post-operative follow-up details.

### Outcomes

The primary outcome was compliance with follow-up MRI surveillance schedules defined in the latest NICE and EANO guidelines (see Supplementary Table 2 for definitions of compliance) [5, 6]. In summary, the NICE guidelines recommend imaging every three to six months post-surgery for two years, and EANO recommend a scheduled scan every three months for two years. If imaging was not completed prior to these dates, a patient was defined as being non-compliant, until the next scan was completed (correlating with time spent compliant to imaging recommendations). The secondary outcomes were OS and PFS. OS was defined as the date of surgery to date of death from any cause. PFS was defined as the time from date of surgery until MRI evidence of tumor recurrence, validated by MDTM agreement. Additional secondary outcomes included initiation of second-line chemotherapy, and re-intervention (repeat surgery or repeat radiotherapy). Follow-up was defined as until date of last clinic appointment, scan (MRI/CT), or date of death. Patients were censored from PFS if they progressed, or death occurred.

### Sample size

The required sample size was derived following a three-center pilot study of 123 patients. The null hypothesis of the study was that more than half of patients (> 50%) will not be scanned in accordance with NICE Guidelines, and that groups scanned according to these guidelines will demonstrate improved OS (superiority assumption), and PFS. This identified a 34% proportion of patients compliant with NICE guidelines, and a median survival of 9.6 months. A hazard ratio of 1.35 was used to set the minimal clinically important difference for the study, which roughly equates to 3 months of OS benefit (similar survival benefit to Temozolomide in Stupp trial). Assuming a mortality rate of 85% of patients during the 24-month follow-up period, to achieve 80% power, with a 5% type 1 error rate, the sample size using a 2 sample, means superiority calculation was 456 patients (approximately 22 Neuro-Oncology units, assuming an average patient list of 20 patients per center).

### Statistical analysis

Data analysis was carried out using R version 4.0.2 using the gpplot2, survminer, survival, and forcats, packages [20–22]. Compliance to NICE and EANO guidelines was summarised using descriptive statistics. This consisted of 3 groups- a group that had every follow-up scan within the recommended time period (Fully compliant); a group that had mixed periods of compliance with imaging, and mixed periods of non-compliance during follow-up (Inbetween); and a group that had no imaging carried out within the recommended timeframe (Never compliant). Fully compliant was further defined as: every scheduled follow-up scan occurring in equal or more frequently than the imaging recommendations (i.e. a patient having three scans, all of which occurred two months apart would be fully compliant). Inbetween was defined as at least one period of time where imaging occurred less frequently than recommended, alongside at least one period of recommended imaging (i.e. a patient who had three follow-up scans, the first two being three-six months apart, followed by a third scan nine months after last scan. Never compliant was defined as having no scheduled imaging within the recommended period (i.e. a patient who had two follow-up scans, both 9 months apart). In the event that a patient had no post-operative imaging, but had survived long enough to have imaging, they were defined as never compliant.

Unscheduled scans were not considered in the compliance definition- if an unscheduled scan occurred, the patient would remain compliant, and the time window for the next scan re-set, as if they had a scheduled scan (i.e. would have 3–6 months to have a further scheduled scan before being considered non-compliant). Continuous variables were analysed using mean (standard deviation [SD]), or median (interquartile range [IQR]) [23]. Differences in characteristics of compliant and non-compliant groups were presented with descriptive statistics. PFS and OS was estimated using Kaplan–Meier survival method. To identify if imaging frequency was associated with increased survival, and to account for any differences in characteristics associated with survival benefit, we conducted multivariable, Cox regression analysis, incorporating compliance as a variable alongside variables known to affect glioblastoma outcomes (age, performance status, extent of resection [EOR], and Stupp protocol treatments) [24–29]. As compliance is not a binary variable, and can change over time (i.e. a patient who is initially compliant with imaging guidelines for the first 12 months of follow-up before becoming non-compliant), compliance was classified as a time varying covariate, with periods of compliance a separate component [30]. This accounts for each individual period spent compliant and noncompliant to imaging, to account for unequal or differential periods of follow-up, minimising bias (i.e. a patient who has spent 20 months compliant and two months non-compliant would be reflected proportionally in analysis). Two separate cox regression models were completed, with compliance according to guideline (NICE and EANO) included separately as a variable in each model (to avoid collinearity). We used Hazard Ratios (HR) and a 95% confidence interval to measure effect size. For each analysis conducted, the number of patients with sufficient data entry was used, giving varying numbers for each analysis point. As biopsy groups have a different scanning recommendation, we carried out additional sensitivity analysis by removing the group, then repeating the analysis.

## Results

### Clinical characteristics

There were 818 patients identified from 26 out of 32 neuro-oncology centers in the UK and Ireland, of which 754 met the minimum data requirement (Table 1). The median number of patients per center was 33 (IQR 17–42) (Supplementary Table 3). Surgical and adjuvant treatments are summarised in Table 1, and detailed in Supplementary Table 4. The median follow-up time after surgery was 10.5 months (IQR 5.3–19.4 months). The total follow-up time was 10,100 (median, 12.5 months/person, IQR 5.2–19.4) person-months of follow-up.

**Table 1:** Baseline characteristics

Characteristic (*N* = 754)	Frequency (%)
Age	
Mean (SD)	60.9 (11.7)
Median [IQR]	63 (54–70)
Range	18–84
Sex	
Male	458 (60.7)
Female	296 (39.3)
Pre-Operative WHO Performance status	
0	302 (40.1)
1	242 (32.1)
2	105 (13.9)
3	30 (4.0)
4	44 (5.7)
Missing	32 (4.2)
Extent of Resection	
GTR	155 (20.6)
STR	386 (51.2)
Biopsy	209 (27.7)
Missing	4 (0.5)
IDH status	
Wild-type	620 (82.2)
Mutant	55 (7.3)
Test failed/not done	62 (8.2)
Missing	17 (2.3)
MGMT	
Unmethylated	349 (46.3)
Methylated	288 (38.2)
Test failed/equivocal/not done	79 (10.5)
Missing	38 (5.0)
Completed Radiotherapy	
Yes	509 (67.5)
No	164 (21.8)
Missing	81 (10.7)
Radiotherapy doses	
60 Gy	250 (33.2)
< 60 Gy	174 (23.1)
None/Unknown	330 (43.7)
Concurrent temozolomide	
Yes	386 (36.3)
No	274 (51.2)
Missing	94 (12.5)
Adjuvant temozolomide	
Yes	346 (45.9)
No	305 (40.5)
Missing	103 (13.6)
Completed Full STUPP protocol	
Yes	112 (14.9)
No	642 (85.1)
Clinical trial enrolment	
Yes	72 (9.5)
No	607 (80.5)
Missing	75 (10.0)

GTR = Gross Total Resection, STR = Subtotal Resection

### Imaging follow-up and compliance

**Fig. 1:**
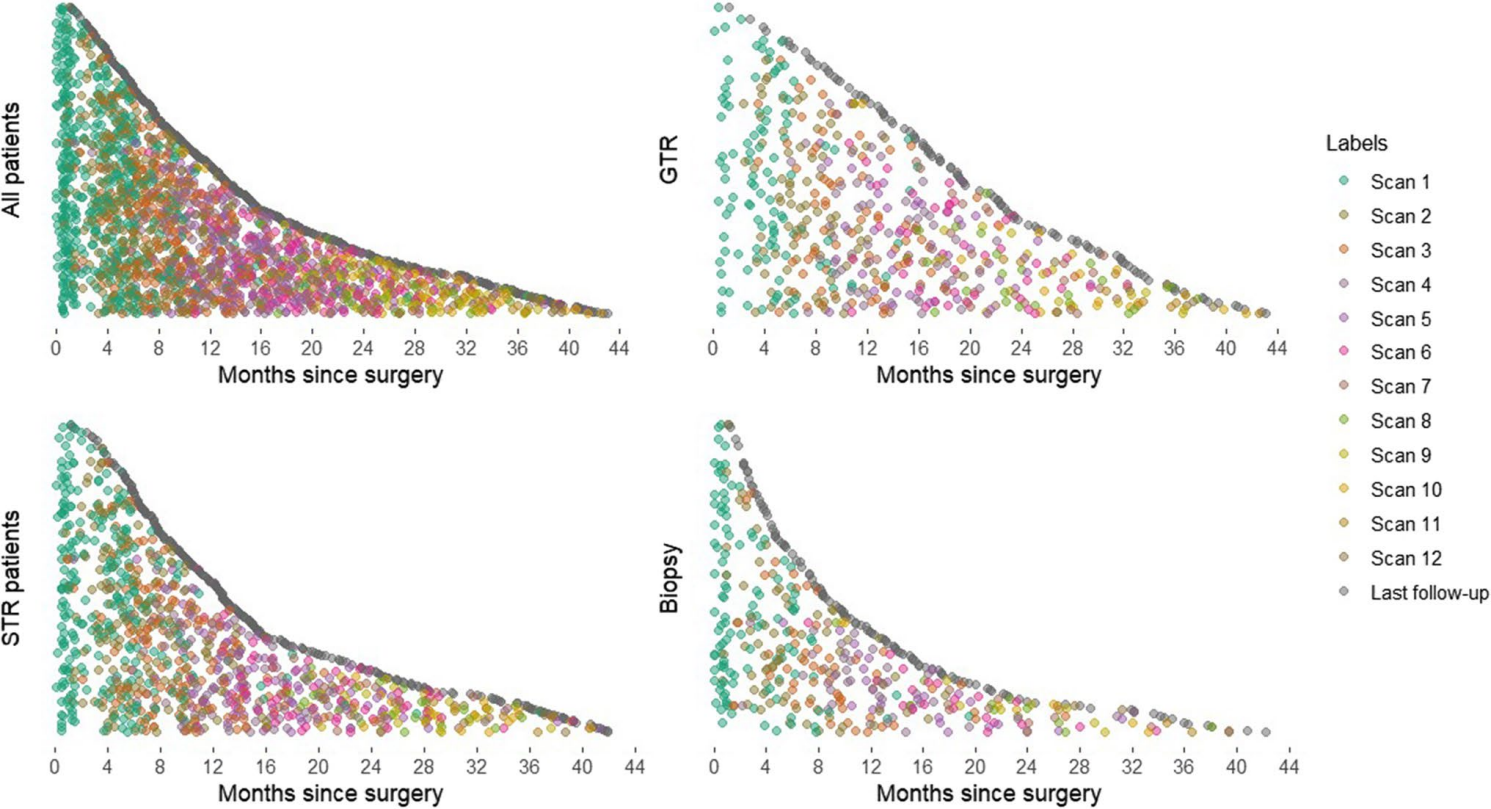
Distribution of MRI scan time following surgery stratified by extent of resection. Each dot represents an MRI scan and each row of dots represents a patient. GTR = gross total resection; STR = subtotal resection

The post-surgical imaging schedules are summarised in Supplementary Table 5 and 6. In total, 522 (69.2%) patients had a post-operative MRI scan within 7 days of surgery. Of these, 462 (88.5%) were a post-operative scan to assess extent of resection, and 60 (11.5%) were for radiotherapy planning without an extent of resection scan. An MRI scan within the 72-h recommended NICE guidelines after resection (excluding biopsy) was seen in 78.0% (*N* = 407/522), and within the 48-h EANO guidelines in 64.2% (*N* = 335/522). The follow-up MRI scan timings after radiotherapy to assess for progression are presented in Fig. 1, and Supplementary Tables 5 and 6. At every follow-up scan, 40–50% showed disease progression. Pseudoprogression was seen in 8.3% of scans (median, IQR 7.8%-9.8%).

**Fig. 2:**
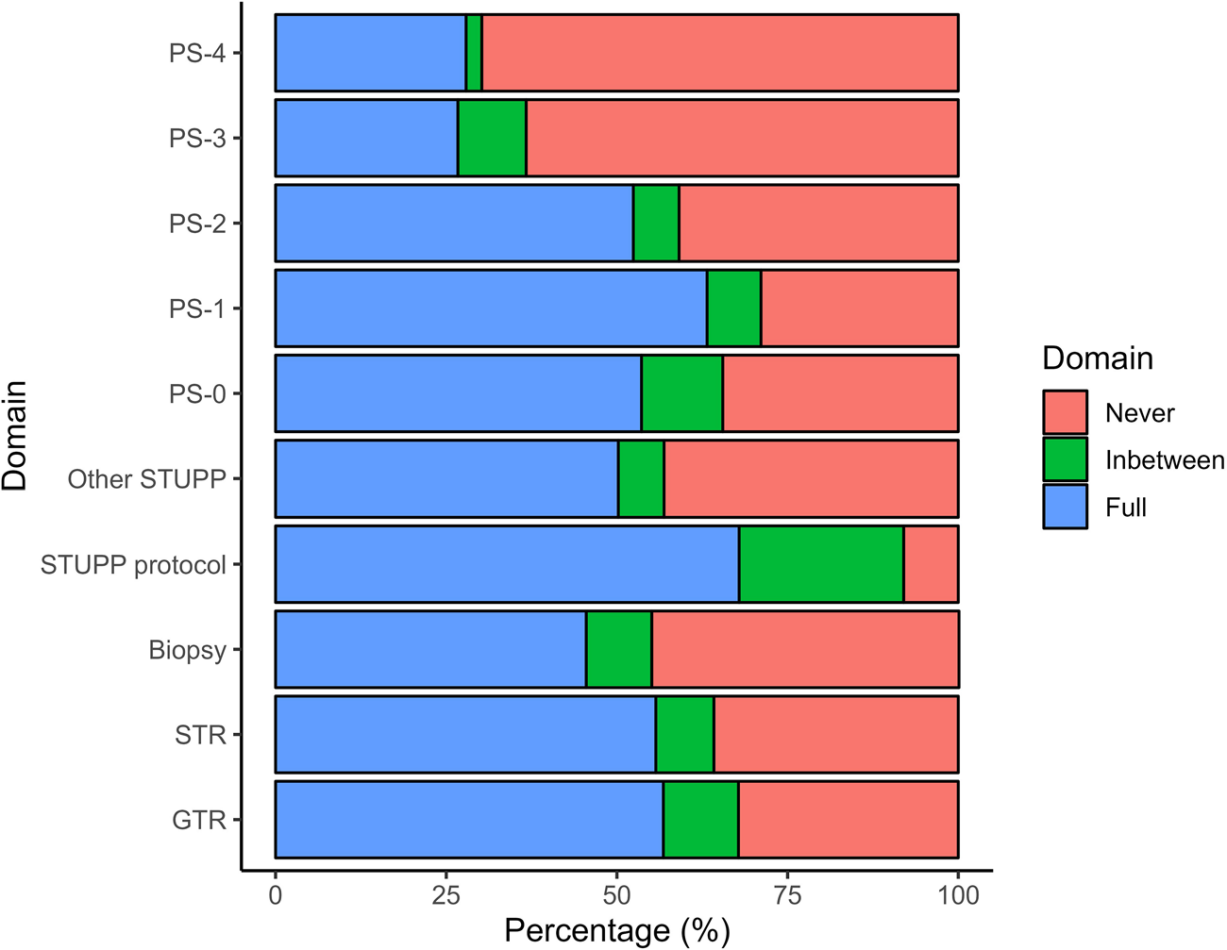
Differences in interval imaging compliance stratified by extent of resection groups, Stupp protocol completion, and WHO performance status (PS). Fully compliant = a group that had every follow-up scan within the recommended time period; Inbetween = a group that had mixed periods of compliance with imaging, and mixed periods of non-compliance during follow-up; Never compliant = a group that had no imaging carried out within the recommended timeframe. STUPP protocol = full completion of STUPP protocol (adjuvant radiotherapy + concomitant and adjuvant chemotherapy); Other STUPP = Any other treatment (partial completion, no treatment)

In total, 398 (52.8%) of patients had imaging intervals that were fully compliant with NICE guidelines, 70 (9.3%) had intervals that moved between recommended and noncompliant scanning, and 286 (37.9%) were never compliant. In total, for the EANO guidelines, 188 (24.9%) of patients were fully compliant; 217 (28.8%) moved between recommended and noncompliant scanning, and 349 (46.3%) were never compliant. Compliance differed depending on EOR, and Stupp protocol completion (Fig. 2, Supplementary Table 7).

### Progression free survival and overall survival

**Fig. 3:**
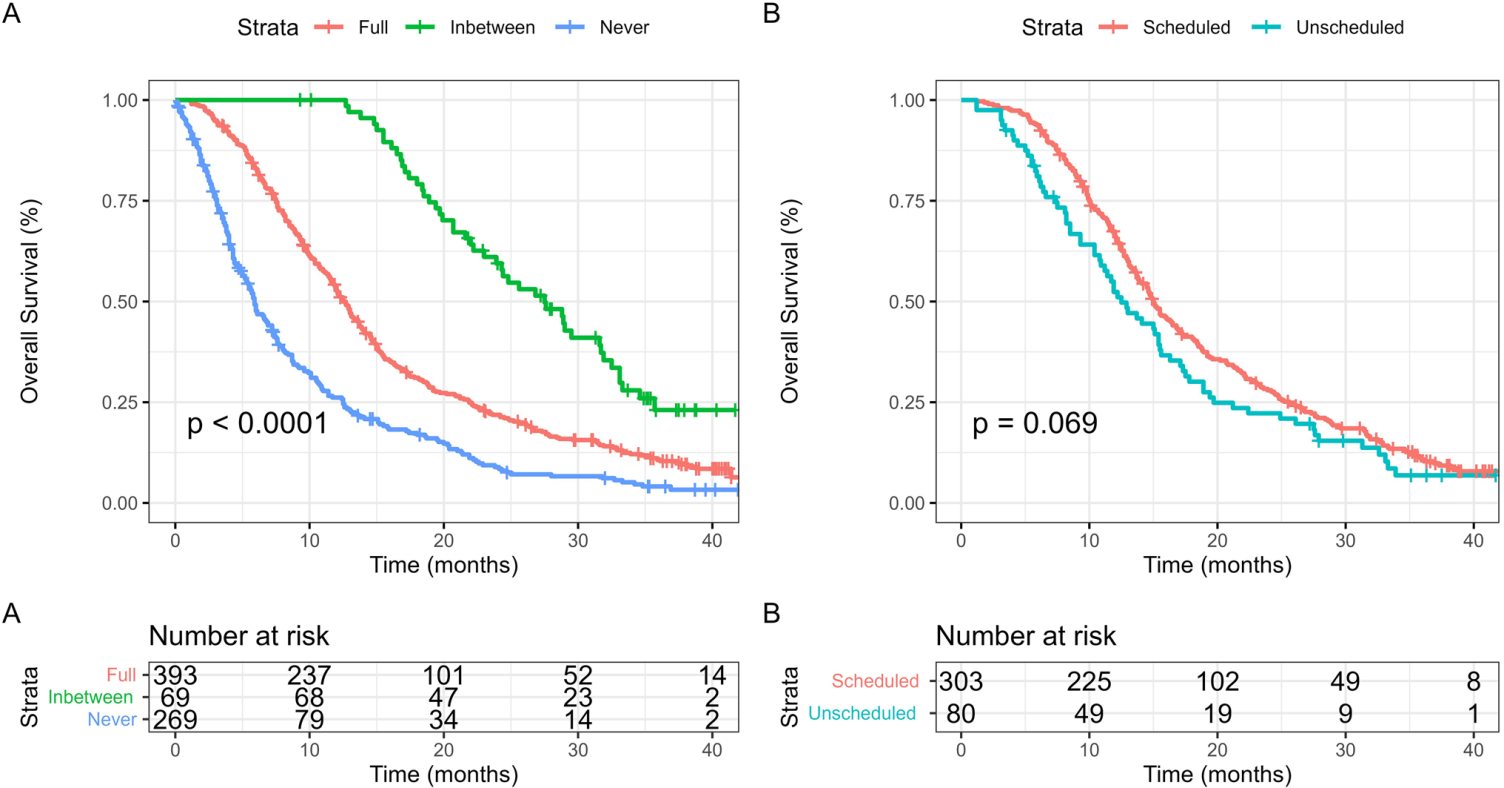
Kaplan–Meier estimates of **A**) OS stratified by NICE imaging compliance (Full, Inbetween, Never), **B**) OS stratified by first progression detected by scheduled vs unscheduled MRI

In total, 684 (90.7%) progressed during the study period. The median PFS was 6.7 months (95% CI 6.2–7.3 months) (Supplementary Fig. 1). 641 patients (85.0%) died during the study period. The median OS using the KM estimate method was 11.4 months (95% CI 10.4–12.4 months), with expected differences depending on EOR, and STUPP protocol adherence (Supplementary Fig. 1). In total, of those that progressed radiologically, 305 (79.0%) had first progression detected using scheduled MRI, with 86 (21.0%) having progression first detected on unscheduled MRI. Of those who died, 305 (47.6%) had progression on MRI before death, with 336 (52.4%) without MRI confirmation of progression. Treatments for progression are shown in Supplementary Table 4. Patients with full and in between compliance were more likely to receive second-line chemotherapy, and re-operation (Supplementary Table 8). Survival differed between the three compliance groups (Fig. 3).

### Association between MRI compliance and outcomes

The multivariable cox regression analysis results are shown in Table 2. On multivariable analysis including age, performance status, EOR, and completion of STUPP protocol, compliance with NICE recommendations (time varying covariate) was associated with increased OS (HR 0.56, 95% CI 0.46–0.66, *P* < 0.001). Compliance with EANO recommendations was also associated with increased OS (HR 0.54, 95% CI 0.45–0.63, *P* < 0.001). Compliance with NICE recommendations was not associated with increased PFS (HR 0.93, 95% CI 0.79–1.10, *P* = 0.349). Compliance with EANO recommendation was not associated with increased PFS (HR 0.99, 95% CI 0.84–1.16, *P* = 0.874). After repeated subgroup multivariable analysis excluding the biopsy group, provided similar results (Supplementary Table 9).

**Table 2:** Multivariable cox-regression analysis of factors associated with OS and PFS

OS			
Variable	Hazard Ratio	(95% Confidence Interval)	*P* value
Age	1.02	1.02–1.03	< 0.001
Performance status 1*	1.20	0.99–1.43	0.0575
Performance status 2	1.07	0.84–1.37	0.5795
Performance status 3	1.16	0.75–1.80	0.5125
Performance status 4	1.08	0.74–1.57	0.6686
Extent of resection- GTR vs Biopsy	0.50	0.39–0.64	< 0.001
Extent of resection- STR vs Biopsy	0.76	0.63–0.92	0.0052
Completed full STUPP protocol	0.52	0.41–0.67	< 0.001
Time spent compliant with NICE imaging recommendations**	0.56	0.46–0.66	< 0.001
Time spent compliant with EANO imaging recommendations***	0.54	0.45–0.63	< 0.001
PFS			
Variable	Hazard Ratio	(95% Confidence Interval)	*P* value
Age	1.02	1.01–1.02	< 0.001
Performance status 1*	1.07	0.89–1.28	0.467
Performance status 2	0.88	0.69–1.12	0.307
Performance status 3	0.84	0.54–1.30	0.438
Performance status 4	0.94	1.18–1.87	0.738
Extent of resection- GTR vs Biopsy	0.52	0.42–0.67	< 0.001
Extent of resection- STR vs Biopsy	0.67	0.55–0.82	< 0.001
Completed full STUPP protocol	0.67	0.53–0.83	< 0.001
Time spent compliant with NICE imaging recommendations**	0.93	0.79–1.10	0.349
Time spent compliant with EANO imaging recommendations***	0.99	0.84–1.16	0.874

*Compared to performance status 0 as reference variable. * Time varying covariate. ** Time varying covariate, different model to presented in table

## Discussion

In this study of patients with glioblastoma, only 52.8% were fully compliant with NICE recommended MRI follow-up schedules, and this was independently associated with an increase in OS compared to those who were not compliant. This study includes real-world data from 26 participating institutions across the UK and Ireland.

Patients in the regular scheduled MRI group had a survival benefit compared to the un-scheduled group. This finding is unexpected, and one possible explanation is that patients who underwent regular imaging had a better pre-operative performance status, were less likely to have a biopsy, and more likely to have completed additional, life-extending treatments [31, 32]. The hypothesis that scheduled MRI will detect asymptomatic disease progression prompting earlier initiation of second line treatments that can stabilise the glioblastoma and increase survival has been extensively debated [9, 33, 34], but remains untested. In our study, patients who had regular scheduled imaging had a higher likelihood of receiving second-line chemotherapy or re-operation, which could support this, but would need to be validated by studies to establish a clear temporal causality- a prospective study to test this hypothesis is unlikely. Reverse causality may affect the association between regular imaging schedule and survival, where patients with better survival are more likely to have regular imaging. Although OS and PFS were longer in the groups who received regular imaging, this could be due to a healthier cohort overall not captured by our baseline characteristics data points. No studies at present have examined this potential effect [35, 36]. Future studies investigating patient fitness in a more comprehensive manner, for example assessing daily activity including steps peri-operatively could provide us better insight on the matter. The finding that the non-full compliant group (Inbetween) had the largest OS by a significant margin, is surprising. This is most likely explained by reverse causality. By nature of having a prolonged follow-up period, due to inherently longer OS, these patients will have had more scans, and were therefore more likely to belong to this group, as opposed to the fully compliant group. Given the median number of post-operative follow-up scans was one, a patient with five or more scans would be more likely to survive longer, but belong to the inbetween category as opposed to the fully compliant group.

The most frequently performed MRI schedule was every 3 months (NICE recommended), although most patients only received one follow up scan in total (excluding the post-operative, and radiotherapy planning MRIs). It is recognised that glioblastoma is incurable for nearly all patients, and that disease progression is inevitable [3]. In our study, most progression occurred after the second follow-up scan after completing radiotherapy and concomitant temozolomide therapy, conducted at 6 months (52.8%). At every follow-up scan, 40–50% showed disease progression.

The patients in this study reflect a broad, less-selected, real-world representation of outcomes for glioblastoma in clinical practice, which explains the shorter OS compared to published trials (11.4 months versus 15–17 months in clinical trial cohorts) [37, 38]. Patients enrolled into clinical trials are more highly selected, often include patients who complete Stupp protocol treatments in full, which may over-estimate GBM survival [39]. Our study included patients who received a biopsy plus any adjuvant radiotherapy and chemotherapy regimes (e.g., short course radiotherapy), which are often excluded from trials, despite being a proportionally larger component of real-world glioblastoma cohorts [26, 40]. This may also explain the difference in pseudoprogression results in our study compared to trials [41].

A study of 277 high grade glioma patients (178 glioblastoma and 99 anaplastic astrocytoma) all treated with maximal surgery, radiotherapy, and temozolomide chemotherapy, proposed optimal timing for MRI monitoring to detect progression in glioblastoma monitoring as, every 7.4 weeks until the end of standard treatment, followed by a gap of 22 weeks, followed by every 27.6 weeks thereafter [10]. As our median time to progression was 6 months (24 weeks), this would mean most patients have progression identified during the inflection period, before the 27.6-week gap. A Cochrane review [7] identified that imaging studies are severely lacking. A single center study reported that having a post-operative MRI scan within 48 h found no association with improved survival- this did not include dynamic follow-up scanning [42]. The GIN-CUP study surveyed national imaging practice, identifying great variability across the UK [11]. MRI surveillance schedule recommendations also vary in international guidelines. For example, NICE recommends 3 monthly for the first 2 years [6], EANO recommends assessing EOR between 24 and 48 h of surgery, and an MRI scan 3–4 weeks after completion of radiotherapy, but a wide interval range of scans every 2–6 months ‘depending on the disease histology' and short-term MRI within 4–8 weeks to confirm disease progression [5]. USA guidelines do not make a formal recommendation for post-operative imaging intervals [5, 43], and instead advocate the incorporation of clinical trial guidelines into clinical practice [43], but these do not reference timing specifically. They do advocate the Response Assessment in Neuro-Oncology (RANO) criteria, but these do not make interval recommendations outside of a moratorium for expecting pseudoprogression [44]. This variation in guidance reflects the paucity of high-quality evidence and a reliance on expert evidence and consensus opinion.

This study has several strengths, and implications for policy. Namely, the study included patients from 26 Neuro-Oncology units and their associated neurosurgical centers in the United Kingdom and Ireland, including over 750 patients with a glioblastoma. This provides evidence from multiple centers, that employ different imaging strategies, increasing generalisability. Importantly, this study provides evidence that regular surveillance in-line with NICE guidance, was independently associated with increased OS, which has not been previously reported [33, 34].

Additionally, this study also describes the association between MRI and OS in a multi-center setting. The study comprises one of the largest glioblastoma cohorts, including data over 6 months from 85% of eligible neuro-oncology centers in the UK and Ireland, with a combined catchment population of 72 million people. The exact progression timeline of glioblastoma has been accurately mapped through this analysis, using the dates of each scan. This indicates that at every 3-month surveillance scan, there is a 40–50% chance of progression.

Furthermore, the results from this study address a key question from the James Lind Alliance Priority Setting Partnerships (PSP)—namely, the association of interval imaging on survival which previous literature has failed to address. The results are useful for guiding patients on likely scan schedules they may face, such as the time length between scans, and scan number. As such, the study findings can inform the development of both imaging studies and trials that investigate the use of imaging protocols on detection of, and initiation of treatments, but also all glioblastoma trials, looking to identify when progression occurs, and the impact of conventional treatments on survival. The implications for policy are that, given the association of regular imaging with improved OS and second-line treatment initiation, regular imaging recommendations should be considered as part of departmental policy/guidelines.

There are several study limitations. Firstly, this was a retrospective, observational study, and therefore does not provide high level evidence [45]. Secondly, the study is representative of UK and Ireland practice, and the findings may not be generalizable to other healthcare settings. Thirdly, there is no agreed definition of ‘compliant’, and the definition used does not permit for realistic unavoidable variation observed in every-day healthcare such as: no shows, waiting lists and machine maintenance. Fourthly, we excluded patients who only underwent biopsy with no adjuvant treatment, which constitutes around 20% of glioblastoma patients. Fifth, the number of records included in each analysis was not homogenous throughout, and there was substantial loss-to follow up data, particularly in adjuvant treatment details (15.8%). We did not correct for this, and for most comparisons, 85% of patients had data available for key outcomes analysed. The magnitude of this effect on our results is unclear.

Furthermore, there was no data retrieved to establish which scans were requested by clinicians but not attended due to patient choice or if too unwell to attend. As such there is a possibility that the irregularly scanned cohort were more likely to be too unwell to attend their appointment and thus, scanning was less frequent. This is supported by Fig. 2, which showcases that compliance declined with worsening pre-operative performance status. The GIN-CUP study highlighted that this could also be because of institutional policies, and that patients are often not scanned due to treating clinician preferences [11].

Finally, our progression definition was defined pragmatically by neuroradiologist interpretation, followed by validation at the neuro-oncology MDTM (tumor board). We did not use RANO defined definitions as these are not used routinely in clinical practice and are mainly used in clinical trials [44]. Therefore, there may be a lack of uniform definitions for pseudoprogression, recurrence, and stable disease in our study.

## Conclusions

In this retrospective, multi-center study, we identified a variation in MRI timing after surgery for glioblastoma in the UK and Ireland. Adherence to MRI follow-up guidelines was low, but was associated with longer overall survival. Prospective studies are needed to investigate the impact of different MRI follow-up schedules, compared to standard practice and/or symptom-directed MRI for the detection of progression, treatment modalities, and overall survival benefit.

## Supplementary Information

Below is the link to the electronic supplementary material.

Supplementary file1 (DOCX 43 KB)

## Data Availability

Study data is available (on reasonable request) by contacting the corresponding authors.
