# Correction to: Imaging timing after surgery for glioblastoma: an evaluation of practice in Great Britain and Ireland (INTERVAL-GB)- a multi-centre, cohort study

**DOI:** 10.1007/s11060-024-04924-8

**Published:** 2025-02-20

**Authors:** INTERVAL-GB Collaborative, Conor S Gillespie, Emily R Bligh, Michael TC Poon, Abdurrahman I Islim, Georgios Solomou, Melissa Gough, Christopher P Millward, Ola Rominiyi, Rasheed Zakaria, Stephen J Price, Colin Watts, Sophie Camp, Thomas C Booth, Gerard Thompson, Samantha J Mills, Adam Waldman, Paul M. Brennan, Michael D Jenkinson, Hidayatul Abdullmalek, Suhaib Abualsaud, Gideon Adegboyega, Chinelo Afulukwe, Najma Ahmed, Michael Amoo, Abdelsalam Nedal Al-Sousi, Yahia Al-Tamimi, Ajitesh Anand, Neil Barua, Harsh Bhatt, Ion Boiangiu, Abbey Boyle, Christiaan Bredell, Talhah Chaudri, Jeremy Cheong, Ana Cios, David Coope, Ian Coulter, Giles Critchley, Harriet Davis, Paolo Jose De Luna, Nayan Dey, Bea Duric, Abdullah Egiz, Justyna O. Ekert, Chinedu Brian Egu, Jinendra Ekanayake, Anna Elso, Tomas Ferreira, Tom Flannery, Kwan Wai Fung, Rahul Ganguly, Sanay Goyal, Emily Hardman, Lauren Harris, Theodore Hirst, Kelvin Sunn Hoah, Sam Hodgson, Kismet Hossain-Ibrahim, Lena Mary Houlihan, Sami Squali Houssaini, Sadid Hoque, Dana Hutton, Mahnoor Javed, Neeraj Kalra, Siddarth Kannan, Efthymia Maria Kapasouri, Andrew Keenlyside, Kristy Kehoe, Bharti Kewlani, Prerna Khanna, Rosaline de Koning, Kunalika Sathish Kumar, Ashvin Kuri, Simon Lammy, Eunkyung Lee, Robert Magouirk, Andrew J Martin, Riccardo Masina, Ryan Mathew, Adele Mazzoleni, Patrick McAleavey, Gráinne McKenna, Daniel McSweeney, Saad Moughal, Mohammad Arish Mustafa, Engelbert Mthunzi, Armin Nazari, Trinh Ton Nu Ngoc, Shiva Nischal, Michael O’Sullivan, Jay J. Park, Anand S Pandit, Jonathan Pesic Smith, Peter Peterson, Isaac Phang, Puneet Plaha, Shyam Pujara, George E. Richardson, Marwa Saad, Shinjan Sangal, Avani Shanbhag, Veekshith Shetty, Natalie Simon, Robert Spencer, Rosa Sun, Irtiza Syed, Jesvin Tom Sunny, Anca-Mihaela Vasilica, Daniel O’Flaherty, Arslan Raja, Daniele Ramsay, Renitha Reddi, Elena Roman, Ola Rominiyi, Dorina Roy, Omar Salim, Jeremiah Samkutty, Jashan Selvakumar, Thomas Santarius, Stuart Smith, Agbolahan Sofela, Edward Jerome St. George, Preethi Subramanian, Vaibhav Sundaresan, Kieron Sweeney, Boon Hoe Tan, Nicole Turnbull, Yuewei Tao, Lewis Thorne, Rebecca Tweedie, Anastasia Tzatzidou, Babar Vaqas, Sara Venturini, Kathrin Whitehouse, Peter Whitfield, Jack Wildman, Isabelle Williams, Karl Williams, Victoria Wykes, Tiffany Tze Shan Ye, Kelvin Sunn Yap, Mahir Yousuff, Asaad Zulfiqar, Neurology, Neurosurgery Interest Group (NANSIG), Soham Bandyopadhyay, Setthasorn Z. Y. Ooi, Abigail Clynch, Oliver Burton, Moritz Steinruecke, William Bolton, Alvaro Yanez Touzet, Hannah Redpath, Seong Hoon Lee, Joshua Erhabor, Orla Mantle, Conor S Gillespie, Emily S Bligh, British Neurosurgical Trainee Research Collaborative (BNTRC), Angelos Kolias, Julie Woodfield, Aswin Chari, Robin Borchert, Rory Piper, Daniel M. Fountain, Michael TC Poon, Abdurrahman I Islim

**Affiliations:** Cambrdige, UK

**Correction to: Journal of Neuro-Oncology (2024) 169:517–529** 10.1007/s11060-024-04705-3

In this article the name of Anand S Pandit was missing in the list of collaborators of the INTERVAL-GB Collaborative.

The original article has been corrected.

