# Supplementary material for: Imaging timing after surgery for glioblastoma: an evaluation of practice in Great Britain and Ireland (INTERVAL-GB)- a multi-centre, cohort study
Source: J Neurooncol. 2024 Aug 6;169(3):517–29. doi: 10.1007/s11060-024-04705-3 (PMC11341661; doi:10.1007/s11060-024-04705-3)
Supplement: Supplementary file 1 — Supplementary file1 (DOCX 43 KB) [file 11060_2024_4705_MOESM1_ESM.docx]

**Supplementary Tables- INTERVAL**

**TEXT SUMMARY**

Supplementary Tables include: PICOS inclusion and exclusion criteria, NICE Guidelines and study definitions, Participants per centre, Adjuvant treatment details, post-surgical imaging characteristics, imaging outcomes for scans 7-12, and subgroup analysis with biopsy group removed.

**Supplementary Table 1: PICOS Criteria**

Table 1: Inclusion and exclusion criteria.

|  | **Include** | **Exclude** |
| --- | --- | --- |
| **Age** | Adults (≥ 18 years) | Children (< 18 years) |
| **Diagnosis** | Histopathology diagnosis of glioblastoma (WHO 2016) | Diagnosis not confirmed by tissue analysis |
| **Date of surgery** | Between 01/08/2018 and 01/02/2019 | Outside of study dates |
| **Type of surgery** | Gross Total Resection*, Subtotal Resection*, Biopsy | Biopsy without adjuvant therapy, no surgical intervention |
| **Adjuvant therapy** | Concomitant and/or adjuvant therapy, or radiotherapy alone with radical intent (e.g., Stupp protocol)[3] | Palliative chemotherapy/radiotherapy |
| **Imaging data** | Information on follow-up MRI employed | No information on follow-up scanning, or follow-up with CT only |
| **Survival data** | Date of death/last follow up if alive available | Date of death/last follow up not available |
| **Completeness of data** | Entries with all of: surgery date, imaging data, and survival data | Entries without all three of: surgery date, imaging data, and survival data. |
| *Gross Total Resection was defined as over 90% of tumour debulking. *Subtotal resection was defined as resection of tumour less than 90%. | | |

**Supplementary Table 2: NICE Guidelines and use in compliance definition for this study.**

| **Scan Type** | **NICE guideline** | **Definition to be compliant** | **EANO guideline differences** |
| --- | --- | --- | --- |
| Post-operative MRI | Consider a baseline MRI scan within 72 hours of surgical resection for all types of glioma. | Not included in compliance definition - due to study objective being follow-up imaging association with OS and PFS | Baseline MRI scan within 24-48 hours. |
| Follow-up imaging | “Possible regular clinical review schedule”  From 0 to 2 years (post-surgery), review every 3 to 6 months. | Scheduled scan completed every 6 months for first 2 years after surgery. Less frequently= noncompliant period, until next scan. | Scheduled scan completed every 3 months for first 2 years after surgery. Less frequently= noncompliant period, Until next scan. |
| Missed scans | Not mentioned in guidelines | If no missed scans and all within time window: compliant. If one or more missed scans (later than recommendation): ‘Inbetween compliant and non-compliant’. No scans performed within time period (later than recommendations) =never compliant. | If no missed scans and all within time window: compliant. If one or more missed scans (later than recommendation): ‘Inbetween compliant and non-compliant’. No scans performed within time period (later than recommendations) =never compliant. |
| Unscheduled scans | Arrange a clinical review, including appropriate imaging, for people with glioma who develop new or changing neurological symptoms or signs at any time. | Unscheduled scans not counted in compliance definition | Unscheduled scans not counted in compliance definition |

**Supplementary Table 3: Participant records uploaded per centre.**

| **Centre** | **Number of patients (%) (N=754)** |
| --- | --- |
| **A** | **37 (4.9)** |
| **B** | **49 (6.5)** |
| **C** | **30 (4.0)** |
| **D** | **36 (4.8)** |
| **E** | **39 (5.2)** |
| **F** | **42 (5.6)** |
| **G** | **11 (1.5)** |
| **H** | **15 (2.0)** |
| **I** | **35 (4.6)** |
| **J** | **7 (0.9)** |
| **K** | **37 (4.9)** |
| **L** | **31 (4.1)** |
| **M** | **50 (6.6)** |
| **N** | **31 (4.1)** |
| **O** | **6 (0.8)** |
| **P** | **30 (4.0)** |
| **Q** | **9 (1.2)** |
| **R** | **29 (3.8)** |
| **S** | **1 (0.1)** |
| **T** | **55 (7.3)** |
| **U** | **3 (0.4)** |
| **V** | **45 (6.0)** |
| **W** | **14 (1.9)** |
| **X** | **56 (7.4)** |
| **Y** | **39 (5.2)** |
| **Z** | **17 (2.3)** |

**Supplementary Table 4: Adjuvant treatment details**

| **Characteristic** | **Frequency (%)** |
| --- | --- |
| **Completed Adjuvant Radiotherapy** | |
| Yes | 509/754 (67.5) |
| No | 164/754 (21.8) |
| Missing | 81/754 (10.7) |
| **Gy** | |
| 60 | 249/509 (48.9) |
| 40 | 79/509 (15.5) |
| 30 | 36/509 (7.1) |
| Other | 60/509 (11.8) |
| Missing | 85/509 (16.7) |
| **Fractions** | |
| 30 | 256/424 (60.4) |
| 15 | 94/424 (22.1) |
| 6 | 25/424 (5.9) |
| Other | 48/424 (11.3) |
| Missing | 1/424 (0.2) |
| **Concurrent TMZ** | |
| Yes | 386/754 (51.2) |
| No | 274/754 (36.3) |
| Missing | 94/754 (12.5) |
| **Adjuvant TMZ** | |
| Yes | 346/754 (45.9) |
| No | 305/754 (40.5) |
| Missing | 103/754 (13.6) |
| **Number of Cycles** | |
| 1-3 | 78/346 (22.5) |
| 4-6 | 150/346 (43.4) |
| >6 | 32/346 (9.2) |
| Missing | 86/346 (24.9) |
| **Second-line chemotherapy** | |
| Yes | 147/661 (22.2) |
| No | 514/661 (77.8) |
| Missing | 0/661 (0.0) |
| Time to second line chemotherapy (months, median) | 10.6 (6.0-17.2) |
| **Agents** | |
| Lomustine | 100/147 (68.0) |
| Bevacizumab | 11/147 (7.4) |
| Other | 36/147 (24.4) |
| Missing | 0/147 (0.0) |
| **Third line chemotherapy** | |
| Yes | 27/134 (20.1) |
| No | 100/134 (74.7) |
| Missing | 7/134 (5.2) |
| Time to third line chemotherapy (months, median) | 15.1 (11.7-21.4) |
| **Agents** | |
| Carboplatin | 6/27 (22.2) |
| Procarbazine | 4/27 (14.8) |
| Lomustine | 4/27 (14.8) |
| Temozolomide | 3/27 (11.1) |
| Other | 10/27 (37.0) |
| Missing | 0 (0.0) |
| **Fourth line chemotherapy** | |
| Yes | 3/27 (11.1) |
| No | 21/27 (77.8) |
| Missing | 3/27 (11.1) |
| Time to fourth line chemotherapy (months, median) | 23.4 (17.8-NA) |
| **Agents** | |
| Lomustine | 1/3 (33.3) |
| Etoposide | 2/3 (66.7) |
| Missing | 0/3 (0.0) |
| **Re-operation** | |
| Yes | 60/754 (8.0) |
| No | 629/754 (83.4) |
| Missing | 65/754 (8.6) |
| Median Time to re-op | 10.8 (3.9-15.6) |
| GTR | 10/60 (16.7) |
| STR | 46/60 (76.7) |
| Biopsy | 3/60 (5.0) |
| Missing | 1/60 (1.7) |
| **Re-irradiation** | |
| Yes | 11/754 (1.5) |
| No | 643/754 (85.3) |
| Missing | 100/754 (13.2) |
| Time to re-irradiation | 21.3 (15.6-25.5) |
| Median Gy (range) | 35 (35-37) |
| Median Fractions (range) | 10 (10-15) |
| TMZ= Temozolomide, GTR= Gross Total Resection, STR= Subtotal Resection. | |

**Supplementary Table 5. Post-surgical imaging characteristics**

| **Characteristic** | **Frequency (%)** |
| --- | --- |
| **Post-operative imaging** | |
| Yes | 522/754 (69.2) |
| Neurosurgical- assess EOR | 462/522 (88.5) |
| Radiotherapy planning | 60/522 (11.5) |
| No | 232/754 (30.8) |
| **MRI within 72 hours*** | |
| Yes | 407/522 (78.0) |
| No | 115/522 (22.0) |
| **Number of follow-up scans after completion of FRT** | |
| 0 | 266/754 (35.3) |
| 1 | 148/754 (19.6) |
| 2 | 83/754 (11.0) |
| 3 | 63/754 (8.4) |
| 4 | 40/754 (5.3) |
| 5+ | 115/754 (15.2) |
| Median (IQR) | 1 (0-4) |
| Range** | 0-13 |
| **Scan indications and outcomes** | |
| **Follow up Scan 1 (N=492)** | |
| Post-op EOR/RT planning scan [Scan 0] to first follow-up scan to detect progression in months[Scan 1] (IQR) | 3.2 (0.9-5.3) |
| Scheduled | 435/492 (88.4) |
| Unscheduled | 57/492 (11.6) |
| - To assess clinical deterioration | 54/57 (94.7) |
| - Other reason/unclear | 3/57 (5.3) |
| Progression | 194/492 (39.4) |
| No progression | 244/492 (49.6) |
| Pseudoprogression | 37/492 (7.5) |
| Missing | 17/492 (3.5) |
| **Scan 2 (N=339)** | |
| Scan 1 to Scan 2 in months (IQR) | 3.0 (2.2-4.0) |
| Scheduled | 283/339 (83.5) |
| Unscheduled | 56/339 (16.5) |
| - To assess clinical deterioration | 55/56 (98.2) |
| - Other reason/unclear | 1/56 (1.8) |
| Progression | 142/339 (41.9) |
| No progression | 152/339 (44.8) |
| Pseudoprogression | 43/339 (12.7) |
| Missing | 2/339 (0.6) |
| **Scan 3 (N=255)** | |
| Scan 2 to Scan 3 in months (IQR) | 2.9 (2.0-3.7) |
| Scheduled | 229/255 (89.8) |
| Unscheduled | 26/255 (10.2) |
| - To assess clinical deterioration | 25/26 (96.2) |
| - Other reason/unclear | 1/26 (3.8) |
| Progression | 113/255 (44.3) |
| No progression | 122/255 (47.9) |
| Pseudoprogression | 20/255 (7.8) |
| Missing | 0/255 (0.0) |
| **Scan 4 (N=194)** | |
| Scan 3 to Scan 4 in months (IQR) | 3.0 (2.3-3.6) |
| Scheduled | 168/194 (86.6) |
| Unscheduled | 26/194 (13.4) |
| - To assess clinical deterioration | 24/26 (92.3) |
| - Other reason/unclear | 2/26 (7.7) |
| Progression | 84/194 (43.3) |
| No progression | 93/194 (47.9) |
| Pseudoprogression | 17/194 (8.8) |
| Missing | 0 (0.0) |
| **Scan 5 (N=154)** | |
| Scan 4 to Scan 5 in months (IQR) | 3.0 (2.1-3.8) |
| Scheduled | 131/154 (85.1) |
| Unscheduled | 23/154 (14.9) |
| - To assess clinical deterioration | 20/23 (87.0) |
| - Other reason/unclear | 3/23 (13.0) |
| Progression | 63/154 (40.9) |
| No progression | 78/154 (50.6) |
| Pseudoprogression | 12/154 (7.8) |
| Missing | 1 (0.7) |

***** Biopsy patients excluded (MRI within 72 hours not recommended for this group). ****** Data collection proforma only included option to input up to 13 scans. EOR= Extent of resection

**Supplementary table 6: Imaging outcomes for scans 6-12**

| **Characteristic** | **Frequency (%)** |
| --- | --- |
| **Scan 6 (N=126)** | |
| Scheduled | 116/126 (92.1) |
| Unscheduled | 10/126 (7.9) |
| To assess clinical deterioration | 10/10 (100.0) |
| Other reason/unclear | 0/10 (0.0) |
| Progression | 60/126 (47.6) |
| No progression | 55/126 (43.7) |
| Pseudoprogression | 11/126 (8.7) |
| Missing | 0/126 (0.0) |
| **Scan 7 (N=89)** | |
| Scheduled | 74/89 (83.1) |
| Unscheduled | 15/89 (16.9) |
| To assess clinical deterioration | 13/15 (86.7) |
| Other reason/unclear | 2/15 (13.3) |
| Progression | 42/89 (47.2) |
| No progression | 43/89 (48.3) |
| Pseudoprogression | 4/89 (4.5) |
| Missing | 0/89 (0.0) |
| **Scan 8 (N=65)** | |
| Scheduled | 60/65 (92.3) |
| Unscheduled | 5/65 (7.7) |
| To assess clinical deterioration | 5/5 (100.0) |
| Other reason/unclear | 0/0 (0.0) |
| Progression | 23/65 (35.4) |
| No progression | 40/65 (61.5) |
| Pseudoprogression | 2/65 (3.1) |
| Missing | 0/65 (0.0) |
| **Scan 9 (N=50)** | |
| Scheduled | 49/50 (98.0) |
| Unscheduled | 1/50 (2.0) |
| To assess clinical deterioration | 1/1 (100.0) |
| Other reason/unclear | 0/1 (0.0) |
| Progression | 23/50 (46.0) |
| No progression | 26/50 (52.0) |
| Pseudoprogression | 1/50 (2.0) |
| Missing | 0/50 (0.0) |
| **Scan 10 (N=42)** | |
| Scheduled | 41/42 (97.6) |
| Unscheduled | 1/42 (2.4) |
| To assess clinical deterioration | 0/1 (0.0) |
| Other reason/unclear | 1/1 (100.0) |
| Progression | 20/42 (47.6) |
| No progression | 20/42 (47.6) |
| Pseudoprogression | 2/42 (4.8) |
| Missing | 0/42 (0.0) |
| **Scan 11 (N=26)** | |
| Scheduled | 20/26 (76.9) |
| Unscheduled | 6/26 (23.1) |
| To assess clinical deterioration | 5/6 (83.3) |
| Other reason/unclear | 1/6 (16.7) |
| Progression | 10/26 (38.4) |
| No progression | 13/26 (50.0) |
| Pseudoprogression | 3/26 (11.6) |
| Missing | 0/26 (0.0) |
| **Scan 12 (N=16)** | |
| Scheduled | 13/16 (81.3) |
| Unscheduled | 3/16 (18.7) |
| To assess clinical deterioration | 3/3 (100.0) |
| Other reason/unclear | 0/3 (0.0) |
| Progression | 9/16 (56.3) |
| No progression | 7/16 (43.7) |
| Pseudoprogression | 0/16 (0.0) |
| Missing | 0/16 (0.0) |

*Data collection proforma only included option to input first 13 scans.

**Supplementary Table 7: Differences in Compliance stratified by Extent of resection, and Stupp protocol compliance.**

| **Group** | **Fully compliant (%)** | **Both compliant and non-compliant (%)** | **Never compliant (%)** |
| --- | --- | --- | --- |
| Gross Total Resection | 88 (56.8) | 17 (11.0) | 50 (32.2) |
| Subtotal Resection | 215 (55.7) | 33 (8.5) | 138 (35.8) |
| Biopsy | 95 (45.5) | 20 (9.5) | 94 (45.0) |
| Completed full STUPP protocol | 76 (67.9) | 27 (24.1) | 9 (8.0) |
| Did not complete full STUPP | 322 (50.2) | 43 (6.7) | 277 (43.1) |

**Supplementary Table 8. Recurrence treatments stratified by compliance category**

| **Treatment** | **Fully compliant (%) [SD]** | | **Both compliant and non-compliant (%)** | | **Never compliant (%)** | | **P value** |
| --- | --- | --- | --- | --- | --- | --- | --- |
|  | **Yes** | **No** | **Yes** | **No** | **Yes** | **No** |  |
| Second-line chemotherapy | 99 (26.7) | 272 (73.3) | 33 (48.5) | 35 (51.5) | 15 (6.8) | 207 (93.2) | **<0.001** |
| Mean time to second-line chemotherapy (months) | 11.5 [7.8] | | 14.9 [9.3] | | 14.3 [13.6] | | 0.162 |
| Third-line chemotherapy | 21 (22.1) | 74 (77.9) | 3 (10.0) | 27 (90.0) | 3 (33.3) | 6 (66.7) | 0.210 |
| Mean time to third-line chemotherapy (months) | 16.0 [8.5] | | 20.0 [6.3] | | 16.6 [5.7] | | 0.742 |
| Fourth-line chemotherapy | 3 (16.7) | 15 (83.4) | 0 (0.0) | 3 (100.0) | 0 (0.0) | 3 (100.0) | 0.094 |
| Mean time to fourth-line chemotherapy (months) | 22.2 [3.9] | | N/A | | N/A | | 1.000 |
| Re-operation | 38 (10.0) | 342 (90.0) | 12 (17.9) | 55 (82.1) | 10 (4.4) | 219 (95.6) | **0.001*** |
| Mean time to re-operation (months) | 10.1 [8.24] | | 12.6 [5.5] | | 8.1 [7.9] | | 0.461 |
| Re-irradiation | 8 (2.2) | 363 (97.8) | 2 (3.0) | 64 (97.0) | 1 (0.5) | 216 (99.5) | 0.203 |
| Mean time to re-irradiation (months) | 19.0 (6.9) | | 25.3 (0.4) | | 20.1 (6.5) | | 0.262 |

**Supplementary Table 9: Subgroup multivariable cox regression analysis of primary outcomes, with biopsy group removed.**

| **OS** | | | |
| --- | --- | --- | --- |
| **Variable** | **Hazard Ratio** | **(95% Confidence Interval)** | **P value** |
| Age | 1.02 | 1.01-1.03 | <0.001 |
| Performance status | 1.01 | 0.93-1.11 | 0.758 |
| Extent of resection- GTR vs STR | 0.64 | 0.51-0.79 | <0.001 |
| Completed full STUPP protocol | 0.46 | 0.35-0.66 | <0.001 |
| Time spent compliant with NICE imaging recommendations* | 0.61 | 0.50-0.75 | <0.001 |
| Time spent compliant with EANO imaging recommendations** | 0.58 | 0.47-0.71 | <0.001 |
| **PFS** | | | |
| **Variable** | **Hazard Ratio** | **(95% Confidence Interval)** | **P value** |
| Age | 1.01 | 1.00-1.02 | 0.004 |
| Performance status | 0.96 | 0.88-1.04 | 0.301 |
| Extent of resection- GTR vs STR | 0.64 | 0.52-0.79 | <0.001 |
| Completed full STUPP protocol | 0.59 | 0.46-0.75 | <0.001 |
| Time spent compliant with NICE imaging recommendations* | 1.08 | 0.89-1.03 | 0.426 |
| Time spent compliant with EANO imaging recommendations** | 1.05 | 0.87-1.27 | 0.598 |
